# POLICY IMPLICATIONS ON CLINICAL PRACTICE: AN EXPLORATION

**DOI:** 10.1093/geroni/igac059.1505

**Published:** 2022-12-20

**Authors:** Danielle Llaneza, Eileen Flores, Lilian Azer, Hanamori Skoblow, Patricia D’Antonio

**Affiliations:** Rutgers University, Piscataway, New Jersey, United States; Penn State, State College, Pennsylvania, United States; University of California, Riverside, Riverside, California, United States; University of Missouri, Columbia, Missouri, United States; The Gerontological Society of America, Washington, District of Columbia, United States

## Abstract

Clinical practice is direct and indirect work with and for patients to improve their wellbeing. Typically, clinical practice is specific to the discipline and institutional body in which providers work. Additionally, caring for older adults requires a review of the biological, psychological, and social aspects of their health to address their needs and diseases. Therefore, policy has significant albeit gradual effects on clinical practice. Health policies and legislation change clinical guidelines, options, and barriers. Federal and state-level policies influence clinical practice factors such as billing, provider knowledge, student training, treatment access, and availability of interventions. Understanding the needs of both patients and providers is imperative to creating clinical practices in line with policy. The collaboration between researchers, providers, and policymakers promotes inclusive and equitable recommendations for health care practices. Yet, often health policy at the federal level is a formidable dynamic that requires expertise and cooperation for all stakeholders that is often not readily accessible. A critical component of this work is reaching providers and patients where they are most often located with information dissemination and outreach efforts. As a GSA policy intern, I explored policy initiatives that directly and indirectly influenced clinical practice across disciplines from the Department of Health of Human Services, National Institute of Aging at NIH, Centers for Medicare & Medicaid Services, and other agencies and reviewed methods that promoted health equity in health care. We need better policy to improve our clinical practice to increase treatment options, expand access, and tailor delivery for our patients.

